# Investigating Chemokine-Matrix Networks in Breast Cancer: Tenascin-C Sets the Tone for CCL2

**DOI:** 10.3390/ijms24098365

**Published:** 2023-05-06

**Authors:** Martha Gschwandtner, Anís N. Gammage, Claire Deligne, Linda F. M. Mies, Alissa Domaingo, Devardarssen Murdamoothoo, Thomas Loustau, Anja Schwenzer, Rupert Derler, Raphael Carapito, Manuel Koch, Matthias Mörgelin, Gertraud Orend, Andreas J. Kungl, Kim S. Midwood

**Affiliations:** 1 Kennedy Institute of Rheumatology, University of Oxford, Oxford OX3 7FY, UK; 2 Institute of Pharmaceutical Sciences, University of Graz, 8010 Graz, Austria; 3 INSERM U1109-MN3T, The Microenvironmental Niche in Tumorigenesis and Targeted Therapy, 67091 Strasbourg, France; 4 University of Strasbourg, 67091 Strasbourg, France; 5 Fédération de Médecine Translationnelle de Strasbourg (FMTS), 67091 Strasbourg, France; 6 INSERM U1109, The Tumor Microenvironment Group, 67091 Strasbourg, France; 7 Laboratoire d’ImmunoRhumatologie Moléculaire, GENOMAX Platform, INSERM UMR_S 1109, Faculté de Médecine, Fédération Hospitalo-Universitaire OMICARE, ITI TRANSPLANTEX NG, Université de Strasbourg, 67091 Strasbourg, France; 8 Institute for Dental Research and Oral, Musculoskeletal Research, Center for Biochemistry, University of Cologne, 50931 Cologne, Germany; 9 Colzyx AB, Medicon Village, Scheeletorget 1, 223 63 Lund, Sweden

**Keywords:** CCL2, MCP-1, tenascin-C, extracellular matrix, breast cancer, myeloid cells, macrophages

## Abstract

Bidirectional dialogue between cellular and non-cellular components of the tumor microenvironment (TME) drives cancer survival. In the extracellular space, combinations of matrix molecules and soluble mediators provide external cues that dictate the behavior of TME resident cells. Often studied in isolation, integrated cues from complex tissue microenvironments likely function more cohesively. Here, we study the interplay between the matrix molecule tenascin-C (TNC) and chemokine CCL2, both elevated in and associated with the progression of breast cancer and playing key roles in myeloid immune responses. We uncover a correlation between TNC/CCL2 tissue levels in HER2+ breast cancer and examine the physical and functional interactions of these molecules in a murine disease model with tunable TNC levels and in in vitro cellular and cell-free models. TNC supported sustained CCL2 synthesis, with chemokine binding to TNC via two distinct domains. TNC dominated the behavior of tumor-resident myeloid cells; CCL2 did not impact macrophage survival/activation whilst TNC facilitated an immune suppressive macrophage phenotype that was not dependent on or altered by CCL2 co-expression. Together, these data map new binding partners within the TME and demonstrate that whilst the matrix exerts transcriptional control over the chemokine, each plays a distinct role in subverting anti-tumoral immunity.

## 1. Introduction

The tumor microenvironment (TME) is populated by unique ecosystems of tumor, stromal and immune cells, surrounded and supported by a disease-specific extracellular matrix (ECM). Molecules within the matrix provide essential biochemical and biophysical cues that orchestrate cell organization and cell–cell communication and directly impact the cell phenotype. Conversations between cells and their surrounding extracellular molecules are important in dictating disease progression and can result in diverse cellular responses from facilitating tumor growth to shaping anti-tumoral immune responses [1,2,3,4]. Whilst often studied in isolation, extracellular cues more likely operate as a cohesive microenvironmental niche. Dissecting integrated tissue-derived signals may therefore lead to a more complete understanding of the role of the tissue microenvironment in tumorigenesis.

Chemokines are small secreted chemoattractants with potent pro-tumoral activities [5,6]. However, targeting these molecules has not met early expectations clinically [7,8]. Within tissues, these “soluble” cues rely on interactions with “insoluble” molecules in the ECM. The best characterized is chemokine binding to glycosaminoglycans (GAGs)/proteoglycans, which are referred to as co-receptors of chemokines. Binding to GAGs immobilizes chemokines in tissues, creating concentration gradients, as well increasing their half-life and inducing their oligomerization, and is essential for binding to and activation of some chemokine receptors [9,10,11,12,13,14,15]. In addition, some studies describe chemokine interactions with other ECM components, such as the chemokine CXCL12 binding to fibronectin [16] and TNC [17] and, CCL21, CXCL13 and CXCL12 binding to collagen IV [18]. However, few studies address the nature of these interactions or how these complexes might signal.

To further investigate chemokine–matrix interplay during tumorigenesis, we examined CCL2 and tenascin-C (TNC). To date, this has only been studied separately in the TME. Both of these molecules are upregulated in many solid tumors, including breast cancer, where high levels are associated with poor prognosis [19,20,21] and metastatic spread [19,22,23,24]. An elevated expression linked to enhanced tumor growth is recapitulated in experimental models of breast cancer, including orthotopic syngeneic grafting models, where TNC or CCL2 expression contributes to disease progression [22,23,24,25,26,27]. CCL2 or monocyte chemoattractant protein-1 (MCP-1), whilst named after its role in attracting monocytes [28,29] also attracts T cells, B cells, NK cells, basophils, macrophages, dendritic cells, myeloid-derived suppressor cells and neutrophils via the seven transmembrane G protein coupled receptor C-C chemokine receptor type 2 (CCR2) [30]. Secreted by tumor cells, fibroblasts and immune cells [31,32], CCL2 promotes breast cancer growth and spread by several means such as inducing the infiltration of inflammatory monocytes and tumor-associated macrophages, by acting as vascular permeability factor or by inducing angiogenesis [22,23,33,34]. More recent studies have also started to explore the role of CCL2 in directly modulating macrophage cell survival, activation and polarization in vitro (reviewed in [35]). Despite a large body of evidence supporting the clear pro-tumoral role of CCL2, the effect of therapeutic anti-CCL2 antibodies in pre-clinical validation ranges from prolongation of survival [22,36], reduction in metastasis [22,37], reduction in infiltrated myeloid cells [23], impacts on the polarization of tumor-associated macrophages [38], to no significant effect on tumor growth and myeloid cell infiltration [39,40], whilst the anti-CCL2 antibody Carlumab (CNTO-888) lacked efficacy in a clinical trial of metastatic castration-resistant prostate cancer as a single agent [41]. TNC is a large ECM protein that is mostly absent in healthy adult tissues but upregulated upon tissue remodeling and inflammation. In cancer, it is highly expressed in the tumor stroma by fibroblasts/myofibroblasts and is organized in tumor matrix tracks [25,42], where it influences a wide range of biology that enables tumor growth and spread, including enhanced vascularization and coordinating immune suppression [17,25,43,44]. Therapeutic antibodies that block TNC-mediated tumor-associated macrophages (TAM) polarization towards a tumor permissive phenotype inhibit tumor growth and block metastatic spread in mouse models [25] but have not yet been assessed in the clinic.

Co-expression of TNC and CCL2 has been reported during cardiac inflammation [45,46,47]. Moreover, a number of studies identify TNC as an inducer of CCL2 expression; recombinant TNC stimulates CCL2 mRNA in human adipocyte culture [48] and TNC overexpression in the heart upregulates CCL2 [46], whilst TNC KO mice exhibit lower levels of CCL2 induction during challenges using models of myocardial infarction [45] and oral squamous cell carcinoma [43]. However, the precise nature of the interaction between chemokine and the matrix remains unclear. Building on the evidence that both TNC and CCL2 individually contribute to breast cancer progression and metastasis, this study aimed to formally examine the co-expression of these molecules and their physical and functional interactions.

## 2. Results 

### 2.1. Co-Expression of TNC and CCL2 in HER2+ Breast Cancer

**Figure 1:**
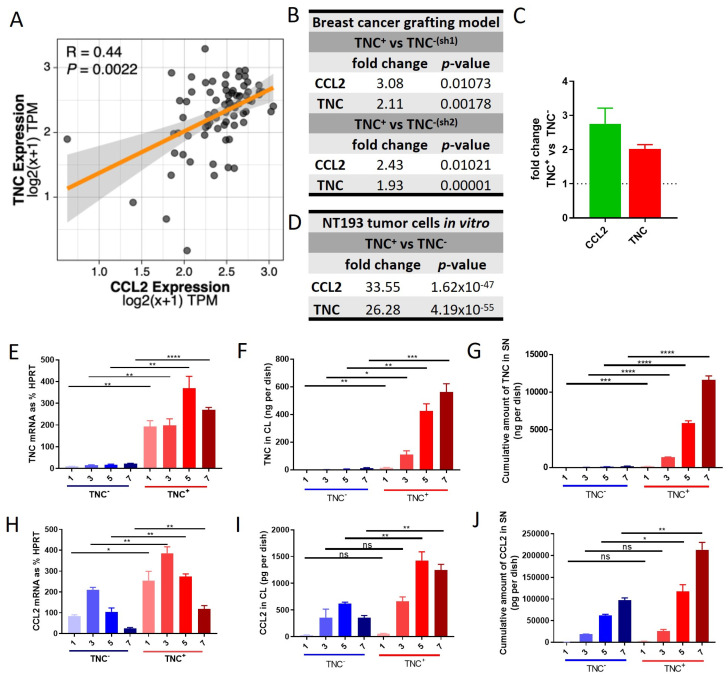
Correlation of TNC and CCL2 expressions in HER2+ breast cancer patients and breast cancer cells in vitro: (**A**) Spearman correlation of CCL2 and TNC expression in the tumor tissue of 77 HER2+ breast cancer patients (BRCA) from The Cancer Genome Atlas (TCGA) RNA-seq dataset. *p* values were adjusted for multiple comparisons with the Benjamini–Hochberg correction (false discovery rate). A linear model was fitted, and the shaded area represents the SEM. Expression units are presented as transcripts per million mapped reads (TPM). (**B**) RNASeq expression data of orthotopic syngeneic breast cancer model in TNC-KO mice using NT193 TNC+ cells and two different types of NT193 TNC− cells for engraftment: TNC−^(sh1)^ or TNC−^(sh2)^. Sh1 and sh2 refer to two different sequences of shRNAs that were used for generating NT193 TNC− cells. Tumors were harvested after 3 weeks. The mean fold change in the expression of CCL2 and TNC in TNC+ cell- versus TNC− cell-engrafted tumors is shown, n = 2 per engrafted cell type and (**C**) graphic depiction of data from (**B**). (**D**) RNASeq expression data of NT193 TNC+ cells and NT193 TNC−^(sh2)^ cells cultivated in vitro. Mean fold change in the expression of CCL2 and TNC in TNC+ cells versus TNC− cells is shown, n = 2 per engrafted cell type. TNC expression in NT193 TNC+ and TNC−^(sh2)^ cells determined on days 1, 3, 5 and 7 on (**E**) mRNA expression level relative to the housekeeping gene HPRT, (**F**) protein level in cell lysate (CL) and (**G**) cumulative protein amount in supernatant (SN). Mean +/− SEM, n = 3. CCL2 expression in NT193 TNC+ and TNC− cells determined on days 1, 3, 5 and 7 on (**H**) mRNA expression level relative to the housekeeping gene HPRT and (**I**) protein level in CL and (**J**) cumulative protein amount in SN. Mean +/− SEM, n = 3. Student’s *t* test was used to compare data sets. **** *p* < 0.0001 *** *p* < 0.001, ** *p* < 0.01, * *p* < 0.05, ns ≥ 0.05.

Tissue levels of both TNC and CCL2 are elevated in people with breast cancer, where the expression of each molecule individually correlates with disease progression [19,20,21,49,50,51]. Here, we focus on our analysis of TNC and CCL2 in HER2+-breast cancer. This disease subtype is particularly associated with a myeloid-rich TME, wherein macrophage abundance is associated with notably poor outcomes and treatment responses [4,52]. To assess any association between the expression of TNC and CCL2, we analyzed RNA sequencing data from 77 HER2+/ERBB2+ breast cancer patients available from The Cancer Genome Atlas. This analysis showed a significant correlation between the expression of these two molecules (Figure 1A). Next, we exploited an in vivo murine model using breast tumor cells lines derived from MMTV-NeuNT mice in which a mutated constitutively active form of rat ERBB2 (NeuNT) is expressed under the control of the mouse mammary tumor virus (MMTV) [17,25]. We analyzed RNAseq data generated from orthotopic syngeneic engraftment of NT193 breast tumor cells in which TNC expression is high (TNC+) or has been knocked down (TNC−). This analysis showed that tumors derived from TNC+ NT193 cells expressed significantly higher CCL2 levels compared to tumors derived from TNC− NT193 cells (Figure 1B,C). In support of these data, RNA seq data of cultured NT193 tumor cells confirmed that CCL2 expression was higher in cells expressing high TNC compared to TNC− cells (Figure 1D). Data from NT193 cells in culture were further validated by an analysis of TNC and CCL2 mRNA and protein over time. As expected, TNC expression was low/undetectable at both mRNA and protein in TNC− cell lines. TNC mRNA expression peaked on day 5 in TNC+ cells and the amount of TNC protein in cell lysate and supernatant increased up to day 7 (Figure 1E–G). In the same cell samples, both CCL2 mRNA and protein expressions were significantly decreased in TNC− compared to TNC+ cell lines (Figure 1H–J). At the mRNA level the expression in TNC− cells was reduced to 35% (day 1), 55% (day 3), 38% (day 5) and 20% (day 7) compared to TNC+ cells, and on the protein level in cell lysates, the CCL2 amount was reduced to 57% (day 1), 51% (day 3), 45% (day 5) and 28% (day 7) and the cumulative CCL2 amount in the supernatant was reduced to 48% (day 1), 75% (day 3), 55% (day 5) and 46% (day 7), indicating both lower and less sustained CCL2 expression in TNC− cells. Together, these data reveal a positive correlation between the expression of TNC and CCL2 in HER2+ breast cancer and show that TNC is required to maintain high levels of CCL2 expression in tumor cells.

### 2.2. TNC Binds CCL2 and the GAG Heparin Interferes with Their Interaction

**Figure 2:**
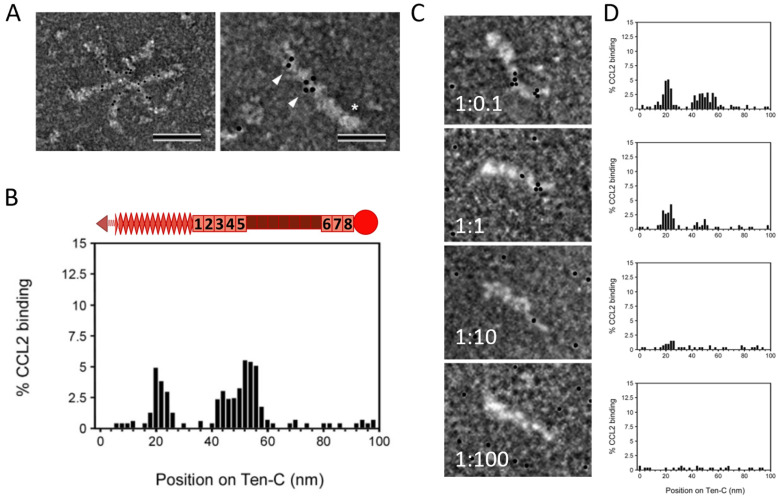
Interaction of TNC with CCL2: (**A**) Transmission electron microscopy using negative staining of gold-labelled mCCL2 binding to TNC hexamers and monomers. Scale bars: 100 nm (left), 50 nm (right). Asterisk shows the FBG domain of monomer, arrowheads point to gold-labelled CCL2. (**B**) Quantification of CCL2 particles bound along the length of TNC on the X-axis. Schematic representation on top of the histogram shows the respective domains of TNC (arrow = assembly domain, check = EGFL repeats, boxes = FN type III repeats, circle = fibrinogen-like globe). (**C**,**D**) Competition of CCL2 binding to TNC with heparin (micrographs and quantification). Scale bars: 50 nm TNC/heparin ratio is shown in micrographs.

The presentation of chemokines by GAGs on the endothelium has been well described [12,13,53], but information about chemokine presentation by other ECM molecules has only recently started to accumulate. TNC is a large ECM protein comprising several distinct domains, namely an assembly domain, epidermal growth factor-like (EGFL) repeats, fibronectin (FN) type III-like repeats and a fibrinogen-like globe. TNC can interact with many different cell surface receptors and matrix molecules, as well as with an increasing number of soluble factors [54]. De Laporte et al. showed that TNC interacts with multiple growth factors, e.g., from the platelet-derived growth factor family or the fibroblast growth factor family, promiscuously via its 5th FNIII repeats domain [55], whilst Loustau et al. (2022) showed that TNC binds to CXCL12 and CCL21 predominantly via its FNIII domains TN3-5 [17,43,56]. In an accompanying paper, we demonstrate that purified recombinant TNC binds to purified recombinant CCL2 in solution using isothermal fluorescence titration (IFT) with a Kd of 2.33 +/− 0.39 µM [57]. When immobilizing recombinant TNC on an SPR-chip and using CCL2 as ligand, a Kd of 10.5 µM was found for this interaction (Figure S2A,B). To further map the binding of CCL2 to the domains of TNC, the interaction was investigated via negative staining electron microscopy. CCL2 was able to bind to hexameric and monomeric TNC (Figure 2A). We did not observe any quantitative or qualitative differences between TNC monomers vs. hexamers in terms of CCL2 binding. We also mapped two major binding sites for CCL2 on TNC, one located within the EGFL repeats and the other in the FNIII repeats (Figure 2B). Binding was abolished by pre-incubation of TNC with increasing amounts of the heparin (mimicking GAGs), with loss of CCL2 binding to FNIII at lower heparin doses in contrast to CCL2 binding to the EGFL repeats. At a molar excess of 100x heparin, all the CCL2 was competed off (Figure 2C,D). Together, these data map two heparin-dependent sites within TNC for CCL2 binding.

### 2.3. Does TNC Impact CCL2-Mediated Chemoattraction?

**Figure 3:**
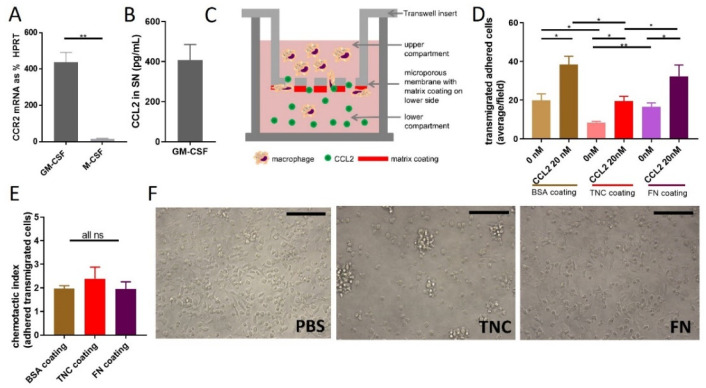
Functional consequences of TNC CCL2 interaction on migration in vitro: (**A**) CCR2 expression of murine bone marrow cells stimulated with M-CSF or GM-CSF for 7 days on mRNA expression level relative to the housekeeping gene HPRT, n = 3, mean +/− SEM. (**B**) CCL2 protein amount determined via ELISA in supernatant of murine bone marrow cells stimulated with GM-CSF for 7 days, n = 5, mean +/− SEM. (**C**) Schematic depiction of transwell migration assay of mBMMs (added to the upper chamber) towards a gradient of mCCL2 (added to the lower chamber). The bottom of the insert was coated with matrix molecules. mBMMs that transmigrated through the membrane and adhered to the bottom of the insert were quantified. (**D**) Transwell migration assay with mBMMs and coating of the bottom surface of the insert with BSA or TNC or FN without chemoattractant or towards 20 nM CCL2. Quantification of cells on matrix coating after 3 h of migration by counting DAPI stained nuclei. Average number of cells/field is shown, n = 3, mean +/− SEM. (**E**) Chemotactic index calculated of 3D. Student’s *t* test was used to compare datasets. ** *p* < 0.01, * *p* < 0.05, ns ≥ 0.05. (**F**) mBMMs seeded on PBS, TNC or FN and imaged after 1 h, 40× magnification, n = 3, representative images are shown. Scale bar is 100 µm.

To understand the functional interplay between TNC and CCL2, we set out to identify the cellular targets of CCL2 within the cell types of the TME. Expression of CCR2, the main CCL2 receptor, in both TNC+ and TNC− NT193 breast tumor cells was below 0.5% of HPRT for all timepoints in both cell types, suggesting that whilst tumor cells are a major source of CCL2 (Figure 1H–J), they are not responding in an autocrine manner to this chemokine. We next focused our analysis on responses within the myeloid compartment, in particular macrophages. These cells are associated with a poor prognosis and treatment response in breast cancer [52], and both TNC and CCL2 play a role in their biology [25,35]. We demonstrated that granulocyte macrophage colony stimulating factor (GM-CSF)-stimulated murine bone marrow-derived macrophages (mBMMs) showed a significantly higher CCR2 mRNA expression than M-CSF-stimulated mBMMs (Figure 3A), which confirmed published expression data [58]. The absolute amount of CCL2 secreted into the supernatant during GM-CSF stimulation over 7 days was relatively low (Figure 3B) compared to the amount of CCL2 that tumor cells secrete (Figure 1J), indicating that additional autocrine stimulation seems unlikely. We therefore chose GM-CSF-stimulated mBMMs for all further assays to model the impact of tumor-derived CCL2 on macrophage behavior in the presence or absence of TNC or a more complex tumor-derived ECM.

One key role of CCL2 is in driving monocyte and macrophage chemotaxis [28,59]. To determine whether TNC impacts CCL2-mediated chemotaxis, we adapted a previously published chemoretention assay setup [17,43], coating the lower side of the Transwell insert with TNC (Figure 3C) to mimic a chemoattractive environment that combines soluble CCL2 with an immobilized TNC matrix. We then analyzed the number of mBMMs that transmigrated from the top well through the membrane towards CCL2 and were retained on the coated lower side of the membrane. Random migration of cells (buffer only in lower well instead of chemokine) served as the control. First, we confirmed that GM-CSF-differentiated mBMMs migrate towards CCL2 in this setup. Transmigration of cells across control bovine serum albumin (BSA)-coated membranes towards CCL2 was significantly higher than random migration in the absence of chemokine. Next, we examined the impact of a matrix substrate on migration. Both random migration and chemokine-induced transmigration were significantly lower on TNC-coated membranes compared to BSA (Figure 3D). To assess whether this reduced migration was specific to TNC or simply caused by coating the membrane with any matrix molecule, we coated membranes with the matrix glycoprotein FN. Both random migration and CCL2-induced transmigration were higher on FN than on TNC and not significantly different to BSA-coated membranes (Figure 3D). The chemotactic index, calculated by dividing the number of cells migrated towards the chemokine by the number of randomly migrated cells, was the same for all three tested coatings (Figure 3E). In line with matrix molecule-dependent effects on cell migration, mBMMs also exhibited distinct morphologies on different substrates. After adhesion for 1h on BSA- or FN-coated substrates, mBMMs showed an elongated shape, whereas on TNC-coated wells, they adopted a rounded shape and formed clusters (Figure 3F). The dependence of cell shape on the matrix coating was also apparent in an adhesion assay with NIH3T3 cells (Figure S1C,E). These data indicate distinct mBMM morphologies induced during adhesion to TNC compared to FN or BSA substrates, and demonstrate specific inhibition of mBMM migration by TNC, which occurred regardless of whether cells were moving randomly or driven to migrate by CCL2-mediated chemotaxis.

### 2.4. Does TNC Impact Atypical CCL2 Functions of Macrophage Survival and Activation?

In addition to orchestrating cell migration, CCL2 has also been reported to exert alternative effects on myeloid cells, such as enhancing their survival/viability or impacting their activation and polarization (reviewed in [35]). The effects of CCL2 on these cell functions were highly context dependent, and so here we firstly set out to examine in our experimental system any role for CCL2 alone on the viability and activation of mBMMs, before analysis of whether the interaction of CCL2 with TNC or with a more complex tumor-derived matrix impacts the action of CCL2 by itself.

**Figure 4:**
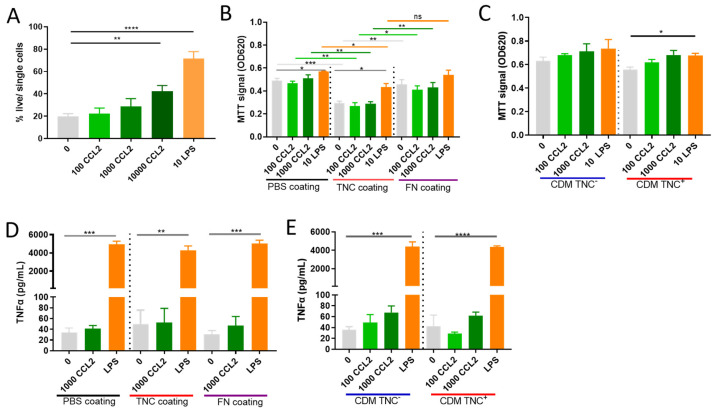
Functional consequences of TNC CCL2 interaction on viability and TNFα secretion in vitro: (**A**) Analysis of CCL2s (100 or 1000 or 10,000 ng/mL) effect on survival of mBMMs in comparison to LPS (10 ng/mL) in vitro in the absence of matrix coating via live/dead staining using flow cytometry. (**B**) MTT survival/proliferation assay of mBMMs seeded on PBS-, TNC- or FN-coated wells (**C**) on cell derived matrix from NT193 TNC+ or TNC−^(sh2)^ cells and stimulated with CCL2 or LPS. (**D**) TNFα level in supernatant of mBMMs seeded on PBS-, TNC- or FN-coated wells or (**E**) on cell-derived matrix from NT193 TNC+ or TNC−^(sh2)^ cells. A student’s *t* test was used to compare datasets. **** *p* < 0.0001, *** *p* < 0.001, ** *p* < 0.01, * *p* < 0.05, ns ≥ 0.05.

Incubation of mBMMs with 100 or 1000 ng/mL CCL2, doses we observed to be secreted by NT193 tumor cells (Figure 1J), had no effect on cell metabolic activity assessed using MTT as a proxy for viability. LPS (10 ng/mL), a positive control known to enhance myeloid cell survival [60,61], significantly increased the viability of mBMMs (Figure 4A, PBS coating). In line with these data, using an orthogonal method to assess cell viability, activation of mBMMs with CCL2 at 100 ng/mL and 1000 ng/mL did not impact survival assessed by live/dead staining and flow cytometric analysis, whilst 10 ng/mL LPS significantly enhanced survival. Supra-physiological doses of CCL2 (10 µg/mL) had an impact on survival, but the effect was substantially less than that induced by LPS (Figure 4B). These data show that CCL2 can induce cell survival but the levels secreted by breast tumor cells do not have a significant effect. 

Next, we assessed the impact of mBMM adhesion to a matrix substrate on CCL2 modulation of cell viability. When cells were seeded on TNC-coated wells, their viability was significantly lower compared to cells seeded on uncoated wells in the absence of CCL2. Additional stimulation with CCL2 on a TNC substrate did not further increase the viability compared to PBS, whereas LPS stimulation significantly improved the viability as expected (Figure 4B, TNC coating). mBMMs coated on a FN matrix did not display any change in viability compared to PBS-coated substrates (Figure 4B, FN coating). Following mBMM adhesion to single matrix substrates, we next generated cell-derived matrices from NT193 tumor cells that express high levels and low/no TNC. When mBMMs were seeded on matrices derived from NT193 TNC+ and TNC-, no significant differences in viability were induced either by the matrix coating or in response to CCL2 stimulation (Figure 4C). 

Finally, to assess the capacity of CCL2 to induce mBMM activation, we examined tumor necrosis factor-alpha (TNFα) secretion as the most widely published response induced by this chemokine [62,63,64]. mBMMs seeded on PBS-coated wells did not induce TNFα secretion upon stimulation with 1000 ng/mL CCL2, in contrast to stimulation with LPS, which potently induced TNFα production, as previously described [65,66,67]. Stimulation of mBMMs adhered to FN-coated or TNC-coated wells with CCL2 also displayed no increase in TNFα production compared to cells that were not treated with chemokine. Similarly, stimulation of mBMMs adhered to complex-tumor-derived CDMs with and without TNC and with CCL2 did not induce TNFα production regardless of the TNC content of the matrix (Figure 4D,E). Together these data show that physiological concentrations of CCL2 do not impact mBMM survival or TNFα production in the assay set-up used here.

### 2.5. TNC Dominates Myeloid Cell Behavior Compared to CCL2 in an In Vivo Breast Cancer Progression Model 

Data from these in vitro cellular models suggest that CCL2 does not affect macrophage survival or activation. However, despite their increased complexity and 3D structure, CDMs are still a simplistic model of the highly intricate TME in vivo. Therefore, we used our orthotopic syngeneic mammary gland grafting model with engineered tumor-cell-derived TNC levels to further investigate (a) whether CCL2 impacts the behavior of macrophages resident within the tumor and (b) whether there is any functional interplay between TNC and CCL2 in vivo. 

First, we confirmed a TNC-dependent phenotype in the orthotopic grafting NT193 breast tumor model. Consistent with published data, engraftment of NT193 TNC+ tumor cells into a wild-type FVB host lead to a significantly increased tumor growth compared to NT193 TNC− tumor cells (Figure S3A) [25]. Secondly, we performed a pilot study using TNC+ tumors with a neutralizing anti-CCL2 antibody, where we injected i.p. at 5 mg/kg and 10 mg/kg (five injections) based on published studies using anti-CCL2 antibodies [68,69,70,71] or i.t. at 1 mg/kg (three injections), which gave a good antibody exposure in this tumor model in our group in the past [25]. The tumor size was smaller when giving anti-CCL2 i.t. compared to i.p. in TNC+ tumors (Figure S3B). An ELISA of the serum collected at the time of sacrifice revealed an increase in CCL2 levels in all anti-CCL2-treated mice compared to untreated mice and we found that the increase in CCL2 was higher in the i.p. treatment schemes (Figure S3C). This phenomenon of increased CCL2 serum levels after anti-CCL2 antibody treatment has already been described in the literature [26,39,72,73] and confirmed that our injections were working as expected. Based on the smaller tumor size with i.t. injections of the elevated serum CCL2, showing that the antibody injections are working as expected and that there is a good exposure to agents via i.t. injections in this model, we selected i.t. injections at 1 mg/kg of anti-CCL2 antibody for further in vivo experiments. This approach also enabled us to mainly target tumor-associated macrophages directly within the tumor and as opposed to the infiltrating monocytes, providing a different point of view compared to previous studies [22,23].

**Figure 5:**
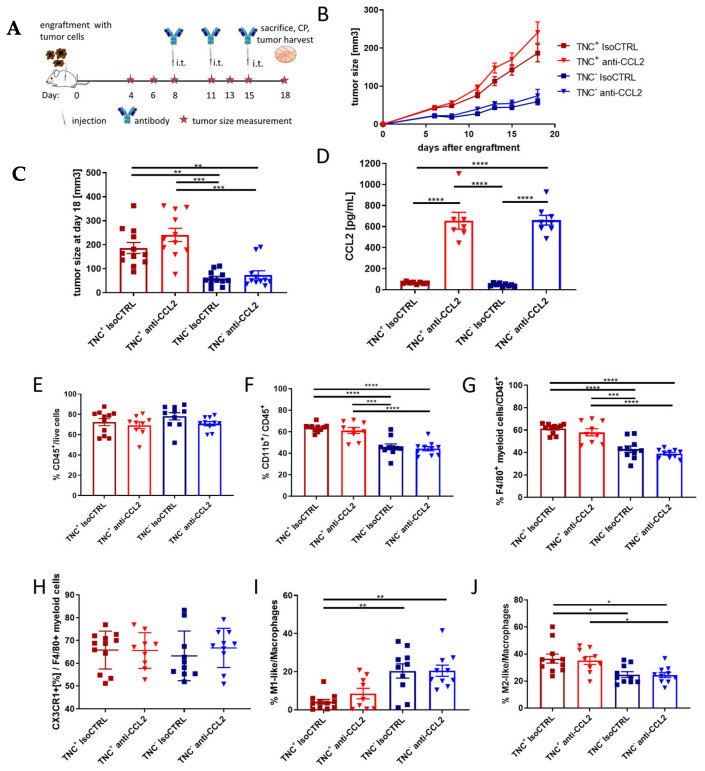
In vivo breast cancer model: (**A**) Timeline of engraftment, tumor size measurements, antibody injections, sacrifice, cardiac puncture (CP) and tumor harvest. (**B**) Tumor size of FVB mice engrafted with NT193 TNC+ (red) or NT193 TNC−^(sh2)^ (blue), treated with anti-CCL2 antibody (triangles) or isotype control (IsoCTRL) (squares) over 18 days and (**C**) on day 18. The data represent four independent experiments and are represented as means +/− SEM. n = 11–12/group. Non-parametric ANOVA and Dunn’s multiple comparisons tests were performed. *** *p* < 0.001, ** *p* < 0.01. (**D**) CCL2 protein in serum samples from cardiac puncture after sacrifice on day 18 determined via ELISA. The data represent three independent experiments and are represented as means +/−SEM. Ordinary one-way ANOVA and Tukey’s multiple comparisons test. **** *p* < 0.0001. (**E**–**J**) Percentage of respective population in TNC+ (red) and TNC−^(sh2)^ (blue) tumors injected with isotype control antibody (IsoCTRL, squares) or anti-CCL2 antibody (triangles). The data represent four independent experiments. n = 9–11/group. In all graphs, means +/− SEM is shown. Ordinary one-way ANOVA and Tukey’s multiple comparisons test or non-parametric ANOVA and a Dunn’s multiple comparisons test were performed, **** *p* < 0.0001, *** *p* < 0.001, ** *p* < 0.01, * *p* < 0.05. All significant differences are shown, otherwise they are ns.

Next, we compared anti-CCL2 treatment with isotype control treatment in mice engrafted either with NT193 TNC− or NT193 TNC+ cells using three i.t injections of anti-CCL2 antibody at a dose of 1 mg/kg (therapeutic treatment scheme Figure 5A). In contrast to the significant impact of tumor-derived TNC on tumor growth, the growth was not significantly impacted by anti-CCL2 treatment (Figure 5B,C) in this model. The ELISA of serum collected at the time of sacrifice revealed an increase in CCL2 in all mice which received i.t. anti-CCL2 compared to isotype-control-treated mice (Figure 5D) independent of the tumor-derived TNC status. We then immune profiled the myeloid compartment of TNC+ and TNC− tumors which had been treated with anti-CCL2 or isotype control antibody to determine the effect of modulating either the matrix or chemokine alone or together. We assessed total CD45+ numbers, total CD11b+ cell numbers among CD45+ cells, monocytes per CD45+ cells, F4/80+ myeloid cells per CD45+ cells, macrophage numbers per CD45+ cells and macrophage polarization status. The number of CD45+ cells per live cell was not affected by either TNC status or anti-CCL2 antibody treatment (Figure 5E). However, in TNC− tumors, significantly fewer CD11b+ and F4/80+ cells among CD45+ cells were observed (Figure 5F,G). There was no difference between the groups in the viability of tumor-derived CD45+ cells (Supplementary Figure S3D). In our model, there was no difference in the percentage of CD11b+ cells, F4/80+ myeloid cells, F4/80+ CX3XR1+ myeloid cells, neutrophils and monocytes in anti-CCL2-treated versus isotype-control-treated tumors (Figure 5F–H and Figure S3E,F). We also could not detect any significant differences in anti-CCL2− versus isotype control-treated groups regarding macrophage polarization towards M1-like (IRF5+) or M2-like (CD206+) macrophages. However, the tumor-derived TNC status had an impact on macrophage polarization, wherein in TNC+ tumors more M2-like macrophages could be found, whereas in TNC− tumors more M1-like macrophages were detected (Figure 5I,J). In summary, tumor-derived TNC dominates the effect on myeloid cell polarization and tumor growth in this model, and treatment with anti-CCL2 antibodies does not markedly impact the tumor myeloid cell compartment.

### 2.6. TNC-CCL2 Gene Interaction Networks Implicate Both Linked and Distinct Roles during Breast Tumor Development In Vivo

**Figure 6:**
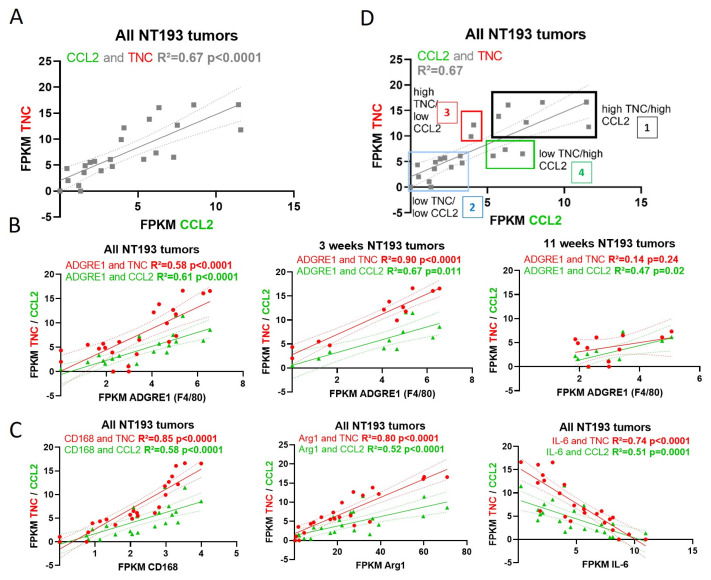

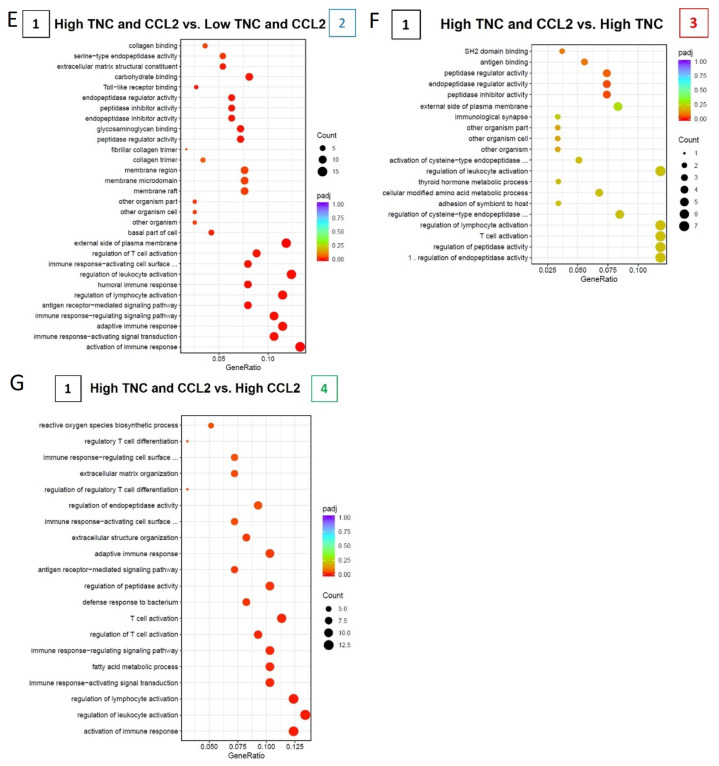
Bioinformatic analysis of the in vivo breast cancer model over time: (**A**) Linear regression curves indicating expression (in fragments per kilobase of transcript per million mapped fragments, FPKM) of TNC in correlation to CCL2 in NT193 tumors at 3 weeks and 11 weeks post-grafting combined. Linear regression curves indicating expression of (**B**) Adgre1 (**C**) CD168, Arg1 and Il-6 in correlation to TNC (in red) and CCL2 (in green), respectively, in all NT193 tumors (3 and 11 week old tumors together) or only 3 week and 11 week old NT193 tumors. *p*-values were determined by Pearson (all tumors analysis) and Spearman (distinctive analysis of 3 and 11 week old tumors) tests. (**D**) From the linear regression curves between CCL2 and TNC in 6A, we separated four groups of tumors depending on their expression status and labelled them 1-4. Gene set enrichment analysis (GSEA) of the differentially expressed genes in (**E**) high TNC and CCL2 tumors compared to low TNC and CCL2 tumors, (**F**) in high TNC and CCL2 tumors compared to high TNC and low CCL2 tumors and (**G**) in high TNC and CCL2 tumors compared to low TNC and high CCL2 tumors. Enriched pathways and over-represented gene sets were selected from Gene ontology database for biological processes, molecular functions and cellular components.

To further examine TNC and CCL2 interaction networks in the in vivo model of breast cancer, we assessed RNAseq data from mice grafted with TNC+ NT193 cells at both the early stage (3 weeks) and a later stage of the disease (11 weeks). These data confirmed a positive correlation of TNC and CCL2 expression across all tumors and at individual time points (Figure 6A and Supplementary Figure S4A). A linear regression analysis revealed that tumor levels of TNC and CCL2 each correlated with myeloid cell markers, including F4/80 (ADGRE1), CSFR1,and CX3CR1, but not with CCR2, with a higher correlation of TNC than CCL2 at the 3 week time point. Interestingly, in late-stage disease, expression of CX3CR1 no longer correlated with either TNC or CCL2, whilst the correlation of CCL2 with CSFR1 and F4/80 increased over time and TNC was not significantly associated with F4/80 at 11 weeks (Figure 6B and Supplementary Figure S4B–E). Moreover, both TNC and CCL2 tumor levels correlated negatively with markers of M1 macrophage status (IL6) and positively with markers of M2 macrophage status (CD168 and Arg1), with a higher correlation of TNC than CCL2 at both 3 and 11 weeks (Figure 6C and Supplementary Figure S5A–C). A co-expression analysis of TNC and CCL2 discriminated four groups of tumors: (1) tumors with overexpression of both TNC and CCL2, (2) tumors with low expression of TNC and CCL2, (3) tumors with overexpression of TNC but not of CCL2 and (4) tumors with overexpression of CCL2 but not of TNC (Figure 6D). A gene set enrichment analysis of genes that were significantly differentially expressed within each tumor group revealed enriched tissue remodeling and matrix organization in TNC/CCL2 high tumors versus TNC/CLL2 low tumors, along with altered innate and adaptive immune processes (Figure 6E). Amongst these pathways, fewer were driven by high CCL2 alone, for example, SH2 binding, amino acid and hormone metabolism, with antigen binding and peptide processing over represented in tumors with high TNC and CCL2 compared to high TNC alone (Figure 6F). Matrix remodeling, reactive oxygen species and fatty acid biosynthesis, and Treg differentiation were specifically associated with TNC and CCL2 high tumors compared to CCL2 high alone (Figure 6G). There was strong conservation of T cell activation shared across both TNC and CCL2 high groups; however, TLR activation, carbohydrate and GAG binding, and humoral immune responses required high expression of both TNC and CCL2 (Figure 6E–G and Supplementary Figure S6A–F). Together, these data support the experimental work in which TNC dominates over macrophage status in the TME, and further indicate both shared and distinct immunological roles for the matrix and the chemokine within the TME.

## 3. Discussion

Our goal in this paper was to dissect any interplay between the matrix molecule TNC and the chemokine CCL2, with a focus on their impact on the myeloid compartment of breast cancer. First, we showed a significant correlation of the expression of these two molecules in HER2+ breast cancer patients. Moreover, we found that TNC expression sustains CCL2 expression in breast tumor cells, both in vivo and in vitro, consistent with a link between these molecules in other disease models and cell types [45,46,47,48]. The regulation of CCL2 expression is complex and context dependent. Our data show that lack of TNC expression causes an increasingly less sustained CCL2 synthesis in NT193 tumor cells. Previous work has shown how murine cardiac fibroblasts transfected with TNC via lentiviral particles upregulated CCL2 compared to GFP transduced cells, and CCL2 secretion was prevented by the TLR4 antagonist CLI-095 [47]. Many proteins impact CCL2 expression in tumor cells (e.g., inflammatory mediators such as interleukin-1, interleukin-6, tumor necrosis factor-alpha (TNFα) or transforming growth factor-β (TGFβ) [74]). This raises the possibility that TNC-mediated induction of CCL2 expression in NT193 cells could occur via TLR4 activation, leading to a direct induction of chemokine expression, and/or indirectly via primary induction of inflammatory cytokines that we know occurs downstream of TNC activation of TLR4 [75]. However, we cannot detect TLR4 at the cell surface by FACS in cultured NT193 cells, suggesting an alternative mechanism at play here. In support of this, TNC purified from early postnatal mice upregulated CCL2 in both WT and TLR4 KO primary murine microglia, demonstrating a TLR4 independent means of CCL2 induction [76]. A truncated and mutated form of TNC (TN-Cfn3/RAA) that is not recognized by RGD-recognizing integrins upregulated CCL2 expression in murine synovial fibroblasts and macrophages isolated from the joints of arthritic mice via integrin alpha 9 [77], highlighting this integrin as a potential candidate receptor, although it is also possible that there exists a cell-type-specific mode of action. 

A companion article [57] identifies the binding of TNC to CCL2, showing the Kd of the interaction to be in a similar concentration range as the interaction of CCL2 with its known co-receptors GAGs/proteoglycans [78,79,80]. In contrast, the affinity of CCL2 for the GPCR CCR2, which is the main signaling receptor for CCL2, is higher (0.77 nM [81]). These findings indicate that not only GAGs/proteoglycans can immobilize and present CCL2 [78,82], but also TNC. We mapped two binding sites within TNC for CCL2, located in the same regions as TNC binding as mapped for other chemokines [17,55]. We also showed that CCL2 binding to both FNIII and EGFL domains was inhibited by heparin, with the EGFL binding demonstrating a higher affinity, suggesting either that CCL2 binds to the same or overlapping locations as heparin binding sites within TNC or that CCL2–heparin complex formation precludes CCL2–TNC binding. Mapping the glycan profiles of purified recombinant TNC from HEK cells reveals one GAG attachment site in the EGF-L repeats and two in TNIII5 [83], implicating these as potential chemokine binding sites and further mapping of specific residues that mediate binary or ternary complexes would be of interest. Moreover, native TNC purified from glioma or milk has distinct modification profiles, notably higher sialated residues and lower mannose glycans compared to HEK-derived TNC [83], suggesting that an in situ analysis of matrix chemokine will also be important to assess.

Turning our attention to any functional interplay, we first examined the canonical role of CCL2 in myeloid chemotaxis. Here, we found that TNC does not impact the chemotactic activity of CCL2, unlike CCL2 binding to GAG co-receptors. We did however note that TNC, but not FN, inhibits the motility of mBMMs, regardless of whether they are moving randomly or towards a stimulus. These findings illustrate the adhesive/anti-adhesive properties of matrix molecules towards different cell types. For instance, TNC anti-adhesive properties have been reported towards endothelial cells or fibroblasts, whereas FN has often been associated with cell adhesive properties in the literature [84,85]. However, adhesive properties have also been reported for TNC and the adhesive/anti-adhesive properties seem to be cell-type-, splice-variant- and context-dependent [86,87,88]. In an adhesion assay performed by Abbadi et al., they found that more mBM-derived M2 macrophages adhered to fibrillar collagen-I-BSA compared to collagen-I-TNC [47]. In our migration setup, more mBMMs adhered on BSA- and FN-coated inserts and significantly less on TNC-coated inserts, pointing towards the anti-adhesive properties of TNC on mBMMs in this setup, without impacting the potency of CCL2 (indicated via the CI) to chemoattract mBMMs. Moreover, the setup of the assay a key consideration. Compared to the setup described in [57], where human monocytes were used as migrating cells and the impact of the interaction of CCL2 and TNC in five-fold molecular excess in solution was investigated, less cells could be detected on the lower side of the membrane when CCL2 was pre-incubated with TNC compared to CCL2 alone. These data highlight a cell- and context-specific effect of TNC on CCL2-mediated migration. 

Aside from CCL2-mediated chemotaxis, intriguing new potential roles for this chemokine are emerging in regulating myeloid cell survival and viability; these data have arisen from a number of different studies using a number of different cell types and are reviewed in [35]. In our study, CCL2 at concentrations secreted by tumor cells did not affect murine mBMM survival or TNFα secretion and an analysis of CCL2s impact on these readouts did not change in the context of different matrix substrates. The lack of an anti-apoptotic/pro-survival effect and lack of an effect on TNFα secretion were previously also shown in the literature [89,90,91] and are in contrast to several other studies that show that CCL2 can induce TNFα production in myeloid cells, such as RAW264.7 macrophages [63], peritoneal exudate macrophages [64] or PMA-stimulated THP-1 cells [62], or that found a positive impact of CCL2 on myeloid cell survival [92,93]. As such, there appears to be both a cell-type- and species-specific response to CCL2, in which our study shows that primary murine BMMs behave differently to cell lines or cells obtained from the peritoneum. In a publication by Li et al. [94], they found that CCL2 at 1000 ng/mL could increase the MTS signal (which is also a tetrazolium dye similar to MTT) in M-CSF-derived human macrophages after only 3 days of incubation and they did not observe a significant effect after 24 h, which matches our findings and suggests that it could be of interest to look at later time points in the future.

Using in vivo targeting of CCL2, we also found that this chemokine had little effect on TAM survival and polarization within the TME. This is in keeping with the treatment regime we used compared to other published studies that describe a reduced recruitment of those cells into the tumor [22,23]. Our observation is likely due to our treatment scheme, where i.t. treatment only started when tumors were palpable; therefore, monocytes/macrophages were already present inside the tumors at the time the treatment began. There was no difference in the percentage of CD11b+ cells, F4/80+ myeloid cells, F4/80+ CX3XR1+ myeloid cells and monocytes in anti-CCL2-treated versus isotype-control-treated tumors. For CCL2, several publications have described a context-dependent potential to influence macrophage polarization towards M1- or M2-like macrophages (reviewed in [35]). In our model, with the described therapeutic treatment scheme we did not detect any significant differences in anti-CCL2 versus isotype control-treated groups regarding macrophage polarization towards M1-like (IRF5+) or M2-like (CD206+) macrophages. However, as previously described [25], the tumor-derived TNC status had an impact on macrophage polarization in the way that in TNC+ tumors, more M2-like macrophages could be found, whereas in TNC− tumors, more M1-like macrophages were detected (Figure 5I,J). 

## 4. Materials and Methods

### 4.1. Cell Lines

The murine mammary cancer cell line NT193 was established from an MMTV-NeuNT tumor [95]. ShRNA was used to generate NT193 cell lines expressing native/ high (TNC+, using lentiviral particles encoding a nontargeting shRNA vector) and low levels (TNC-, using lentiviral particles expressing an shRNA vector specific for tenascin-C) of tenascin-C [24]. NT193 cells were cultured in DMEM high glucose with Glutamax (Gibco, Waltham, MA, USA) containing 10% heat-inactivated fetal bovine serum (Gibco), 100 U/mL penicillin–streptomycin (Gibco) and 10 µg/mL puromycin (Sigma-Aldrich (St. Louis, MO, USA)). The cells were cultivated at 37 °C and 5% CO_2_ in a humidified atmosphere. Cells were periodically checked for tenascin-C expression via real-time PCR (as described below) and for the absence of Mycoplasma (LookOut^®^ Mykoplasma kit, Sigma). 

### 4.2. TNC and CCL2 mRNA and Protein Profiling in NT193 Cell Lines

TNC+ and TNC− NT193 cell lines were harvested using Trypsin EDTA (Gibco) and seeded at a concentration of 5 × 10^4^ cells/mL in 3.6 mL growth medium in 60 mm TC-treated dishes (Falcon) and cultivated at 37 °C and 5% CO_2_ in a humidified atmosphere. For each timepoint (harvest at days 1, 3, 5 and 7), one dish of each cell line (TNC+ and TNC− NT193) was prepared. For longer time points, the medium was removed and replaced with fresh medium at days 3 and 5. Cell seeding and cultivation were repeated three independent times. 

For the protein analysis, the supernatant was collected and stored at −20 °C until use. Cells were washed with ice cold PBS and lysed on ice using ice cold RIPA lysis buffer (50 mM Tris-HCl pH 7.4, 1% Triton-X 100, 0.5% Na-deoxycholate, 0.05% SDS, 150 mM NaCl, 2 mM EDTA) with protease inhibitor cocktail (protease-inhibitor cocktail 1, Fisher, 1280–1640). The expressions of TNC and CCL2 were analyzed by ELISA. All ELISAs were performed according to the manufacturer’s instructions (Tenascin-C Large (FNIII-B) Assay Kit (IBL, 27767), Mouse CCL2/JE/MCP-1 DuoSet ELISA (R&D Systems, Minneapolis, MN, USA, DY479-05)), developed with KPL TMB peroxidase substrate (KPL, 50-76-02) and peroxidase substrate solution B (KPL, 50-65-02) and read on a FLUOstar Omega (BMG Labtech, Aylesbury, UK). The fitting of the standard curves and quantification of samples was performed using MARS Data Analysis Software, Version 3.10 R6 (BMG Labtech). To normalize the results of the total protein concentration in cell lysates from NT193 cells, a bicinchoninic acid (BCA) assay (Thermo Fisher (Waltham, MA, USA), 23227) was performed according to the manufacturer’s instructions. 

For the mRNA analysis, cells were washed with PBS and lysed in RLT lysis buffer (RNeasy Mini Kit, Qiagen (Venlo, Netherlands)) containing 1% β-mercaptoethanol on ice and harvested with a scraper and vortexed. Total RNA was isolated using an RNeasy Mini kit (Qiagen). The concentration and purity of the isolated RNA was determined using a NanoDrop One (Thermo Fisher). A total of 500 ng RNA was reverse transcribed into cDNA using a High-Capacity cDNA Reverse Transcription Kit (Applied Biosystems™ (Waltham, MA, USA)). The real-time PCR reaction was performed in a 384-well plate setup and read on a ViiaTM7 Real-Time PCR system (Thermo Fisher). A TaqMan Universal PCR Master Mix (Thermo Fisher) and the following TaqMan primers (all Thermo Fisher) were used in triplicate: murine HPRT (hypoxanthine guanine phosphoribosyl transferase) Mm00446968_m1, murine CCL2 Mm00441242_m1, murine CCR2 Mm01216173_m1 and murine TNC Mm00495662_m1. Data were analyzed using the ΔCt method in ViiaTM7 software, v1.6.1 and shown as a percentage expression of HPRT.

### 4.3. TNC and CCL2 mRNA Profiling in Syngeneic Grafting In Vivo Model

The in vivo model, preparation of the sequencing library and sequencing and data processing have been described previously [17]. In short, the tumor cell engraftment with 1 × 10^7^ NT193 cells (TNC+ or TNC− (sh1TNC and sh2TNC; sh1 and sh2 refer to two different sequences of shRNAs that were used for generating NT193 TNC− cells [24])) was performed upon sedation of female TNC KO FVB mice in the surgically opened left fourth mammary gland that was surgically closed afterward. At the endpoint (here 3 and 11 weeks) tumors were frozen in liquid nitrogen. The sequencing library was prepared from mRNA derived from the tumors with the Ion Total RNA-Seq Kit v2 (Thermo Fisher Scientific). Sequencing was performed on an ion proton sequencer with the Ion PI™ Hi-Q™ Sequencing 200 Kit (Thermo Fisher Scientific (Waltham, MA, USA)). The transcriptome data were processed by the RNASeqAnalysis plugin from the Torrent Suite Software 5.06 (Thermo Fisher Scientific) [17]. For all gene expression data, dysregulated genes were selected based on a *p*-value or adjusted *p*-value (*p* < 0.05) cutoff of 10% and a minimum log2 fold change of +/− 0.8. From the normalized data of 3 and 11 week tumors, simple linear regression analyses with Pearson or Spearman correlation tests were performed between TNC, CCL2 and other genes of interest significantly dysregulated. After distinguishing tumors with a high expression of TNC, CCL2 or both, a GSEA analysis was performed between each condition by using WEB-based Gene Set AnaLysis Toolkit (WebGestalt) based on the Gene ontology functional database for biological processes, molecular functions and cellular components. 

### 4.4. TNC and CCL2 mRNA Profiling in Human Tumor RNA Sequencing Data

Bulk RNA sequencing (RNA-seq) data of 77 HER2-enriched primary breast tumors from The Cancer Genome Atlas (TCGA) were retrieved from the Genomic Data Commons via the TCGA biolinks package in R (version 2.9.4; [96]). Transcripts were quantified and normalized by the original authors [97] using the RSEM algorithm [98], and these values were multiplied by 106 to provide transcripts per million mapped reads (TPM). TPM values were log2(x + 1) transformed prior to analysis. A pairwise correlation of TNC and CCL2 was performed using the Spearman rank test, and *p* values were adjusted for multiple comparisons with the Benjamini–Hochberg correction (i.e., false discovery rate, FDR).

### 4.5. Recombinant Proteins

His-tagged human TNC (hTNC) was purified according to published protocols [99,100] and an additional lipopolysaccharide (LPS) removal washing step using 0.1% Triton-X 114 during nickel column purification was included. Murine strep-tagged TNC (mTNC) was purified according to published protocols [43]. Purified TNCs underwent an in-house quality control (Supplementary Materials and Supplementary Figure S1). Purified human plasma fibronectin (hFN) was provided by Dr. Wing to [101]. Mouse CCL2 (mCCL2) was obtained from PeproTech (250-10). The following protein combinations were used for in vitro studies based on protein availability: transmission electron microscopy: mTNC and mCCL2, surface plasmon resonance: hTNC and hCCL2, transwell migration assays: hTNC, hFN and mCCL2 and mBMM activation assays: hTNC, hFN and mCCL2. A comparison between the sequences of hTNC and mTNC is described in [57]. The QC of recombinantly expressed hTNC and mTNC was performed via SDS-PAGE and Commassie blue staining as well as an adhesion assay with fibroblasts (Figure S1A–E).

### 4.6. Gel Electrophoresis, Coomassie Blue Staining, Western Blot

For the quality control of hTNC and mTNC, sodium dodecylsulfate polyacrylamide gel electrophoresis (SDS-PAGE) was performed, and for hTNC, Western blotting was also performed, both as described in [99]. A NuPAGE^TM^ 4–12% BisTris gel (Thermo Fisher) was used. An amount of 1.5 µg or 100 ng of purified protein was loaded per well for Coomassie blue staining or Western blotting, respectively. For the Western blot, the anti-TNC MAB1911 (Merck Millipore, Burlington, VT, USA) at 1/1000 and anti-mouse immunoglobulins HRP from rabbit (Dako) in blocking buffer were used for detection.

### 4.7. Adhesion Assay

The adhesion assay was adapted from a protocol published in [99]. Cell culture plates (24 well) were coated with hTNC, mTNC or hFN at a coating concentration of 1 µg/cm^2^ at 4 °C o/n. The uncoated surface was saturated with 1% BSA. Uncoated wells were included as control.

A total of 1 × 10^5^ NIH3T3 fibroblasts (Sigma) per well were seeded in DMEM high glucose (Sigma) with 10% FBS (Gibco) and 1% Pen/Strep (Gibco) and cells were imaged after 3 h with an Olympus CKX-41 inverted microscope at 20× magnification. 

### 4.8. Endotoxin Test

Endotoxin testing of in-house purified proteins was performed using the PyroGeneTM Recombinant Factor C kit (Lonza, Basel, Switzerland).

### 4.9. Transmission Electron Microscopy

A visualization of the interaction of TNC with CCL2 was performed via negative staining and transmission electron microscopy as previously described [43]. Briefly, TNC samples (20 nmol/L) were incubated with a 3 molar excess of CCL2 labelled with 5 nm colloidal gold for 1 h at 37 °C in TBS pH 7.4 and gold particles along the TNC monomer or hexamer, starting from the TA region (0 nm on the X-axis) to the FBG domain (100 nm on X-axis), were analyzed. For competition experiments, TNC was pre-incubated with different molar excesses of heparin (TNC:heparin = 1:0.1, 1:1, 1:10, 1:100, Heparin Sigma-Aldrich, B9806) for 1h at 37 °C. Specimens were examined with a Philips/FEI CM 100 TWIN transmission electron microscope operated at a 60 kV accelerating voltage. Images were recorded with a side-mounted Olympus Veleta camera with a resolution of 2048 × 2048 pixels (2 K × 2 K) and the ITEM acquisition software version 7.0. Binding of CCL2 particles to TNC was determined by counting the number of gold particles along the length of the TNC monomer. The number of molecules from 500 randomly picked distinct TNC molecules was determined.

### 4.10. Surface Plasmon Resonance (SPR)

For the coupling of TNC to the NTA chip (BR-1004-07, GE) using its His-Tag, the NTA Reagent Kit (GE, 28-9950-43) and a Biacore X100 (GE Healthcare, Uppsala, Sweden) were used. The chip was conditioned with regeneration buffer (1350 mM EDTA, 60 s, 10 µL/min) and washed with 3 mM EDTA (60 s, 10 µL/min). NiCl_2_ solution was flushed over the active flow cell only (60 s, 10 µL/min) followed by a 0.17 µM TNC solution (60 s, 10 µL/min).

The coupling was verified by an increase in RU units. The binding of CCL2 was tested using a concentration range of 0–20 µM in PBS + 0.005% Tween20. The Kd values were calculated by blotting the RU against the concentration and applying a steady state fit using the Biacore evaluation software version 2.0.

### 4.11. Bone Marrow Cell Isolation, Differentiation and Culture

Murine bone marrow cells were isolated by flushing out the bone marrow of the femur and tibia of naïve wild-type FVB mice (8–16 weeks old) and lysing red blood cells [102]. A total of 1 × 10^7^ cells were differentiated into murine bone-marrow-derived macrophages (mBMMs) in growth medium (RPMI1640 + L-Glutamine (Gibco, 21875-034), 10% heat-inactivated FBS (Gibco, 10500-064), 1% Pen/Strep (Gibco, 15140-122), 10µM β-Mercaptoethanol (Gibco, 21985-023) with 50 ng/mL granulocyte-macrophage colony-stimulating factor (GM-GSF, PeproTech, 315-03) or macrophage colony-stimulating factor (M-CSF, PeproTech, 315-02)). After 7 days, adherent cells were collected by rinsing with ice-cold PBS or lysed directly in the dish for subsequent RNA isolation to investigate CCL2, CCR2 and TNC expressions as described above. 

### 4.12. Migration Assay of mBMMs

Transwell inserts with a 5 µm pore size (Corning (Corning, New York, NY, USA), 3421) were coated on the lower surface at 1 µg/cm^2^ with matrix molecules for 1h at 37 °C, washed with PBS, blocked overnight in 1% BSA in PBS at 4 °C and washed again in PBS before use. An amount of 600 µL migration medium (RPMI 1640 + 0.1% BSA) with or without 20 nM CCL2 (a concentration well established for migration assays with CCL2 [78]) was added to the lower chamber. Murine bone-marrow-derived macrophages (generated as described above) at 1 × 10^6^ cells/mL in 100 µL migration medium were added to the upper chamber and incubated for 3 h at 37 °C and 5% CO_2_. The medium was then aspirated from the inserts and cells were removed from the upper side of the membrane with cotton buds and PBS washing. The transmigrated mBMMs on the lower side of the membrane were fixed in 4% formaldehyde and stained with DAPI. Each membrane was imaged at 5 positions (middle, top, bottom, left and right) at 40× magnification with an Olympus CKX4.1. The cells were counted using ImageJ software, version 1.53f51. The chemotactic index (CI) was calculated by dividing the number of cells that migrated towards the chemokine by the number of randomly migrated cells in the absence of chemokine.

### 4.13. mBMM Activation Assay

#### 4.13.1. Activation in the Absence of Matrix Molecules

mBMMs were isolated as described above and 1.5 × 10^5^ cells in 200 µL were seeded per well and left to settle for 3 h at 37 °C and 5% CO_2_. One hundred stock solutions of LPS (Sigma, L2630) or CCL2 were then added to each well to reach a final concentration of 10 ng/mL (LPS) or 100 ng/mL and 1000 ng/mL (CCL2) and the plate was incubated for 24 h. Then, the plate was either used directly for an MTT assay or the supernatant was collected for a TNFα-ELISA (mouse TNF-alpha DuoSet ELISA (R&D Systems, DY410)).

Activation in the presence of individual coated matrix molecules:

To mimic the interaction of CCL2 with individual matrix molecules and potential subsequent cell activation, plates with coated matrix molecules were generated. Therefore, matrix molecules were coated at 1 µg/cm^2^ overnight at 4 °C and wells were washed with PBS before use. mBMMs were seeded onto the plates and stimulated with CCL2 and LPS as described above.

#### 4.13.2. Activation on Cell Derived Matrices (CDMs)

The protocol for the generation of CDMs was adapted from published protocols [99,103,104] and performed in 96-well plates with chemicals from Sigma-Aldrich unless stated otherwise. The wells were coated with 1% gelatin for 1 h at 37 °C, crosslinked with 1% glutaraldehyde for 20 min at room temperature (RT) and stopped with 1M glycine for 30 min at RT. Washing steps with PBS were performed after each step. NT193 TNC+ or TNC−^(sh2)^ cells were treated with mitomycin-C solution for 30 min at 37 °C and 2.6 × 10^5^ cells were seeded in 300 µL per well. After 24 h, 200 µL medium per well was exchanged with growth medium containing 50 µg/mL L-ascorbic acid. The medium was exchanged every 2 days and cells were cultured for 18 days. Cells were washed very gently with PBS and 200 µL of prewarmed extraction buffer (20 mM NH_4_OH, 0.5% Triton-X-100 in PBS) was added for 30 min at 37° and left overnight at 4 °C. Microscopically it was confirmed that all cells were lysed. The next day, the CDMs were washed with PBS and left for an overnight washing step in PBS at 4 °C. To digest DNA, the CDM was incubated twice with 200 µL of DNase I (Roche, Basel, Switzerland, 11284932001) at 100 µg/mL diluted in DMEM for 1 h at 37 °C to ensure complete removal of DNA from residual cells and washed thrice with PBS. mBMMs were seeded onto the plate containing the CDM and stimulated with CCL2 and LPS as described above. 

MTT assay: 10% of the total volume MTT (3-(4,5-dimethylthiazol-2-yl)-2,5-diphenyltetrazolium bromide) reagent (Thermo Fisher Scientific, M6494) was added to the cell medium. The plate was incubated for 2 h at 37 °C and 5% CO_2_. Then, 50% of the total volume solubilization buffer (10% SDS in 0.01M HCl) was added and incubated overnight at 37 °C and 5% CO_2_. The optical density at 620 nm was read with an FLUOstar Omega (BMG Labtech).

### 4.14. In Vivo Procedures

All animal procedures were carried out in accordance with the UK Animals (Scientific Procedures) Act 1986 and with the University of Oxford (Clinical Medicine) Ethical Committee approval (P4BEAEBB5, 21 August 2022), or for mice housed at INSERM, according to the guidelines of INSERM and the ethical committee of Alsace, France (CREMEAS). The agreement number was D 67-482-033 at the animal facilities of INSERM U682 and U1109, and mice were kept under pathogen-free conditions in cages providing disposable homes and nesting paper, together with food and water at discretion. Wild-type FVB mice were obtained from Charles River Laboratories.

### 4.15. Tumor Engraftment and Antibody Treatment

Immune competent wild-type female FVB mice at the age of 8–14 weeks were used for the orthotopic engraftment experiments. NT193 TNC+ and TNC−^(sh2)^ cells were freshly thawed two weeks prior to each engraftment. After anesthetizing, shaving and disinfecting the area, the mice were injected with 1 × 10^7^ NT193 TNC+ or TNC−^(sh2)^ cells in sterile PBS (total volume 50 µL) into the left fourth mammary gland using an insulin syringe (BD Medical, 324892, Franklin Lakes, NJ, USA). As the engraftment takes place into a wild-type host, host-derived TNC is still present and the terms “NC+” and “TNC−” refer to tumor-derived TNC. The tumor size was measured every 2–3 days using a caliper. The tumor volume was calculated according to the formula: volume = (length × width × height)/2. 

The therapeutic anti-CCL2 antibody (InVivoMAb anti-mouse/human/rat CCL2, Clone 2H5, BioXCell) or respective isotype control (InVivoMAb Armenian hamster IgG isotype control, Clone PIP, BioXCell) was injected intra tumorally (i.t.) at a concentration of 1 mg/kg on days 8, 11 and 15 after engraftment. In the pilot study, an intra peritoneal (i.p.) injection of the therapeutic antibody was also tested at 5 mg/kg and 10 mg/kg on days 1, 4, 8, 12 and 15 after engraftment. Mice were sacrificed on day 18 after engraftment. Immediately after sacrificing, the mice blood was collected from the heart with a syringe and a 25 gauge needle. Blood was allowed to clot at 37 °C for approximately 20 min, spun down and the serum was collected for use in CCL2 ELISAs. Tumors were collected and processed directly for analysis by flow cytometry as described below. Four independent experiments were performed, each containing mice representing all treatment groups. The timeline of the in vivo experiment is visualized in Figure 5A.

### 4.16. Flow Cytometry

Freshly harvested tumor tissue was cut into small pieces and digested in dissociation medium (DMEM high glucose with Glutamax (Gibco) containing 5% heat inactivated FBS (Gibco), 500 µg/mL Liberase TM (Roche), 100 μg/mL DNase I (Roche)) for 30 min at 37 °C under agitation. Cells were separated using a 70 μm cell strainer (Falcon) and treated with a flow cytometry buffer (PBS with 5% FBS and 2 mM EDTA). Surface and intracellular staining was performed according to standard protocols. Briefly, 2 × 10^6^ cells were blocked for 15 min with TruStain FcXTM (BioLegend, San Diego, CA, USA) and then stained with a surface antibody mix for 25 min. All surface antibodies were obtained from Biolegend and used at 1/200 dilution, only anti-CCR2 was obtained from R&D Systems and used at 1/25 dilution. The surface antibodies (used in two panels) were anti-Ly-6C-Brilliant Violet 785 (128041), anti-Ly-6G-Brilliant Violet 650 (127641), anti-CD45-PE-Cy7 (103113), anti-CD11b-PerCP-Cy5.5 (101227), anti-F4/80-Pacific Blue (123123), anti-CD206-Alexa Fluor 700 (141733) and anti-CX3CR1-Phycoerythrin (149005). Dead cells were stained using a LIVE/DEAD™ Fixable Yellow Dead Cell Stain (ThermoFisher). For intracellular staining, cells were subsequently treated with fixation/permeabilization solution and BD Perm/Wash™ Buffer (BD Biosciences) before staining for 30 min with anti-IRF5-Phycoerythrin (IC8447P-025, R&D Systems) at 1/100 dilution. Stained cells were detected with an LSR II flow cytometer (BD Biosciences). Data analyses were performed using FlowJoTM software version 10. Cell populations were defined as follows: monocytes: CD45+ CD11b+ Ly6C+ Ly6G+ (medium), neutrophils: CD45+ CD11b+ Ly6C+ Ly6G+ (high), F4/80+ myeloid cells: CD45+ CD11b+ F4/80+ and macrophages: CD45+ CD11b+ F4/80+ CX3CR1+. Staining for IRF5 was used to denote M1-like macrophages and CD206 M2-like macrophages.

### 4.17. Statistical Analysis

The results were plotted and analyzed using GraphPad Prism 9.5.0 software. The Gaussian distribution was tested via the d’Agostino–Pearson test if the sample size was high enough (>8). An unpaired t test or one-way ANOVA with Tukey’s multiple comparisons test was used to determine the significance of differences from Gaussian datasets. Otherwise, the Mann–Whitney test or nonparametric ANOVA with Dunn’s multiple comparisons were used. 

## 5. Conclusions

In summary, tumor-derived TNC dominates the effect on myeloid cell polarization and tumor growth in the in vivo and cellular models of breast cancer that we have used in this study, as well as controls CCL2 transcription in tumor cells. These data raise the intriguing question of why TNC interacts with CCL2 at all. Is this merely a non-specific, non-functional binding of a soluble chemokine to a “sticky” matrix molecule? Or does this matrix–cytokine combination play a role yet to be uncovered? It was recently shown that the matrix tracks are different in the early stages of this cancer model examined here compared to late-stage disease [105], where TNC plays a more important role in track maturation and macrophage abundance between 3 and 11 weeks. It is possible that the TNC/CCL2 interplay influences end stage tumor biology rather than early stage. Indeed, interrogation of RNA seq data at different stages of tumor development points to a continued association of the chemokine and the matrix during disease progression, but within which the chemokine–myeloid link becomes stronger over time in contrast to the matrix. Moreover, it could be that a CCL2-rich TNC matrix affects the BMM phenotype beyond the primary outputs we have examined here, or in fact on the immune axis beyond the myeloid compartment. A bioinformatic analysis points to a requirement for high levels of both TNC and CCL2 with enriched binding to both glycan moieties, as well as with tumor-infiltrating B cells and/or antibody-mediated immune responses. A more complete picture of these novel chemokine–matrix interaction networks may be useful in understanding why current drugs targeting chemokines in cancer have failed and in the development of new strategies in this area. 

## Data Availability

Data are available upon request.

## Supplementary Materials

The supporting information can be downloaded at: https://www.mdpi.com/article/10.3390/ijms24098365/s1.
