# Supplementary material for: Investigating Chemokine-Matrix Networks in Breast Cancer: Tenascin-C Sets the Tone for CCL2
Source: Int J Mol Sci. 2023 May 6;24(9):8365. doi: 10.3390/ijms24098365 (PMC10179296; doi:10.3390/ijms24098365)
Supplement: Supplementary file 1 [file ijms-24-08365-s001.zip › ijms-2348760-supplementary.pdf]

Supplementary file

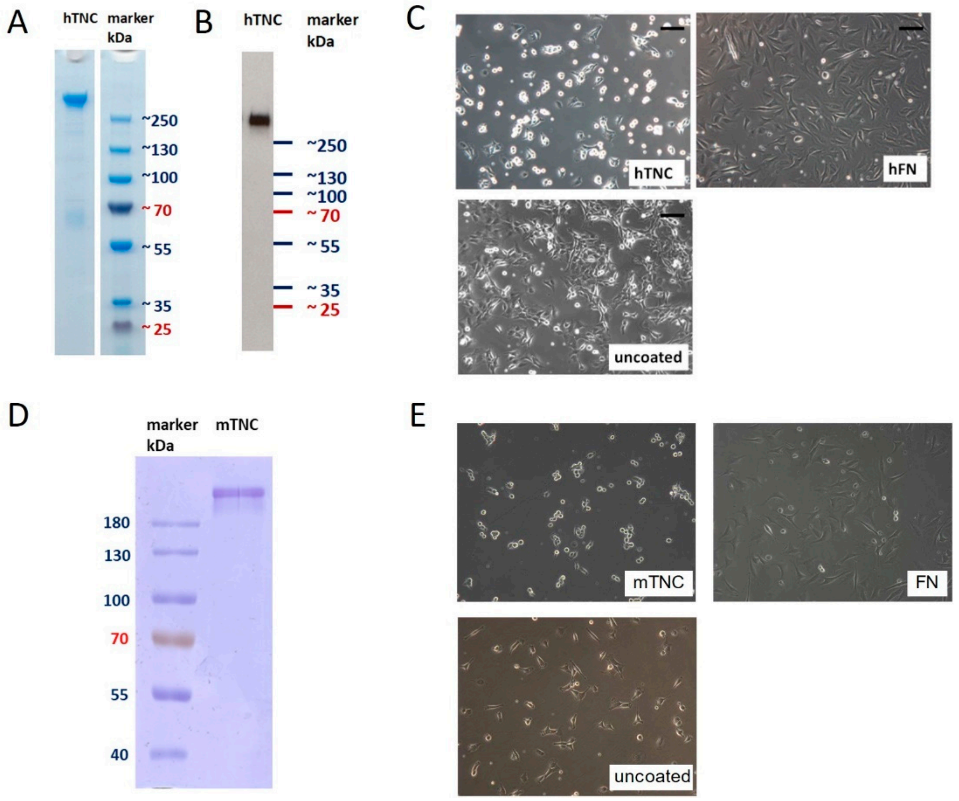

Supplementary figure S1: QC of hTNC and mTNC: S1A) SDS-PAGE and subsequent Coomassie blue staining of hTNC in comparison to PageRuler™ Plus Prestained Protein Ladder. S1B) SDS-PAGE and subsequent Western Blot of hTNC detected with anti-TNC MAB1911 in comparison to PageRuler™ Plus Prestained Protein Ladder. S1C) Adhesion assay of NIH3T3 cells. Phase contrast images taken 3 hours after seeding on different types of matrix coating at 1µg/cm2. Scale bar is 100 µm. Representative images are shown. S1D) SDS-PAGE and subsequent Coomassie blue staining of mTNC in comparison to PageRuler™ Prestained Protein Ladder. S1E) Adhesion assay of NIH3T3 cells. Phase contrast images taken 3 hours after seeding on different types of matrix coating at 1µg/cm2. Representative images are shown.

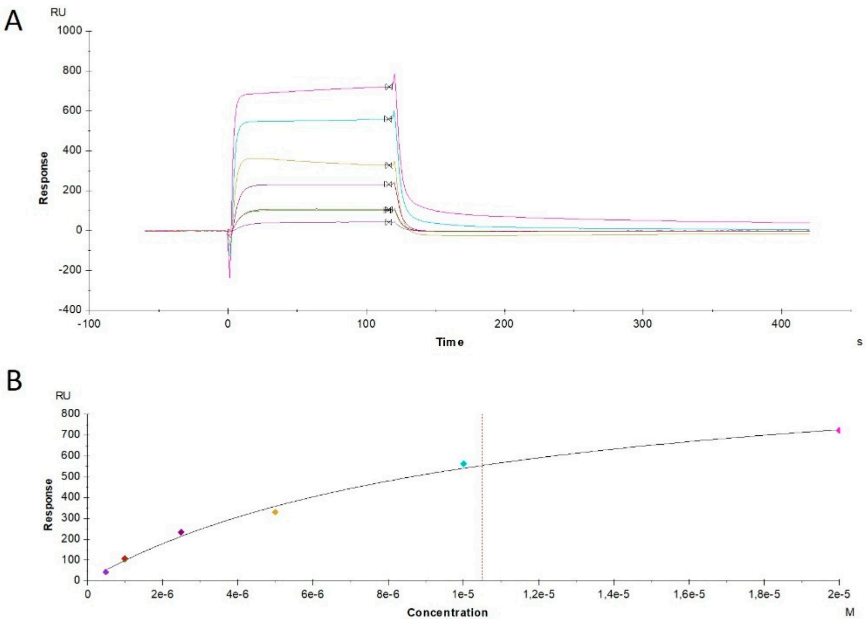

Supplementary figure S2: Interaction of TNC with CCL2 investigated via SPR: S2A) Sensogram of CCL2 binding to TNC immobilized on an NTA-chip. S2B) Blotting the RU at steady state from S2A against the respective CCL2 concentration and applying a steady state fit using the Biacore evaluation software.

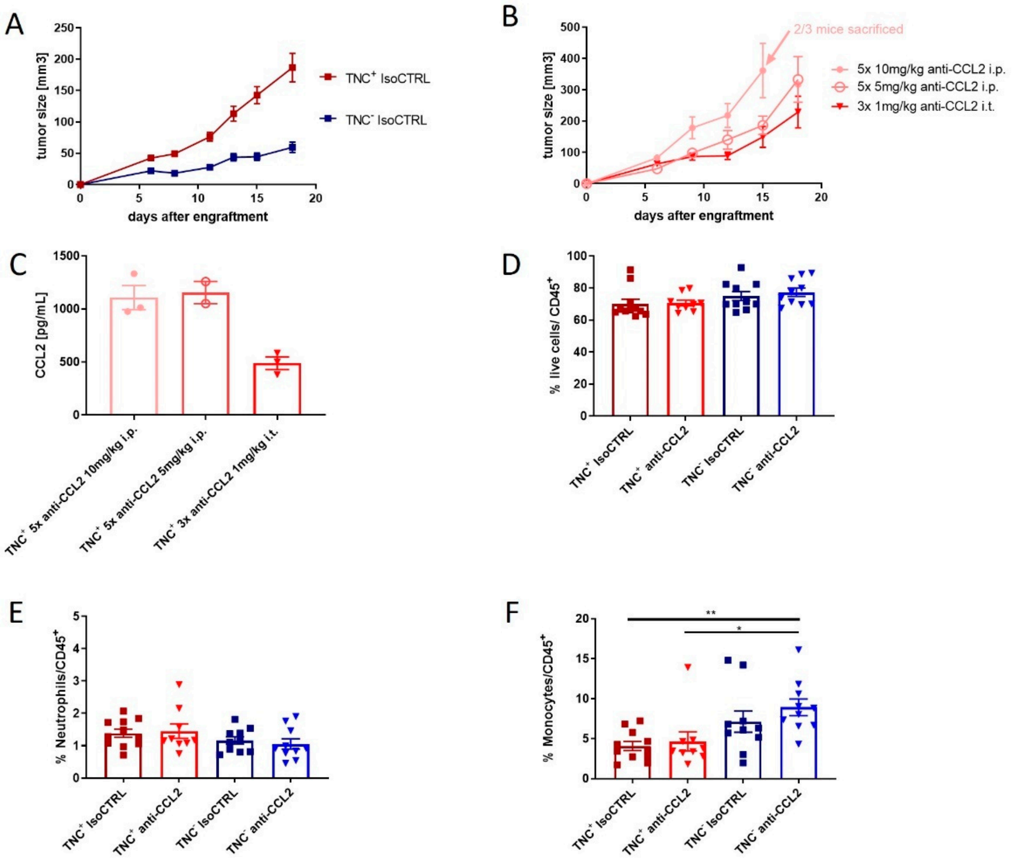

Supplementary figure S3: Pilot study of *in vivo* breast cancer model and additional flow cytometric analysis of myeloid cells from in vivo breast cancer model: S3A) Tumor size of FVB mice engrafted with NT193 TNC+ (red) or NT193 $TNC^{-(sh2)}$ (blue), treated isotype control antibody (IsoCTRL) (squares) over 18 days. Data represent four independent experiments and are represented as mean +/- SEM. n = 11-12/group. Non-parametric ANOVA and Dunn's multiple comparisons test. ***P< 0.001, **P<0.01. S3B) Engraftment with TNC^{+} NT193 tumor cells and three different treatment schemes with anti-CCL2 antibody (intra tumorally (i.t.). at a concentration of 1mg/kg on days 8, 11 and 15 after engraftment (triangles) or intra peritoneally (i.p.) at 5mg/kg (filled circle) and 10mg/kg (empty circle) on days 1, 4, 9, 12, 15 after engraftment. On day 15 2/3 mice in the group 5x 10mg/kg i.p. had to be sacrificed due to one dimension of the tumor reaching the maximum tolerable length. Data represent one experiment. n=2-3/group. Mean +/- SEM. S3C) CCL2 protein in serum samples from cardiac puncture after sacrifice of mice from (S2B) determined via ELISA. Mean +/- SEM. S3D) Percentage live cells of respective population or 3E) neutrophils of respective population S3F) monocytes of respective population in TNC^{+} (red) and $TNC^{-(sh2)}$ (blue) tumors injected with isotype control antibody (IsoCTRL, squares) or anti-CCL2 antibody (triangles). Data represent four independent experiments. n=9-11/group. In all graphs mean+/- SEM is shown. Ordinary one-way ANOVA and Tukey's multiple comparisons test or non-parametric ANOVA and Dunn's multiple comparisons test. ns ≥ 0.05.

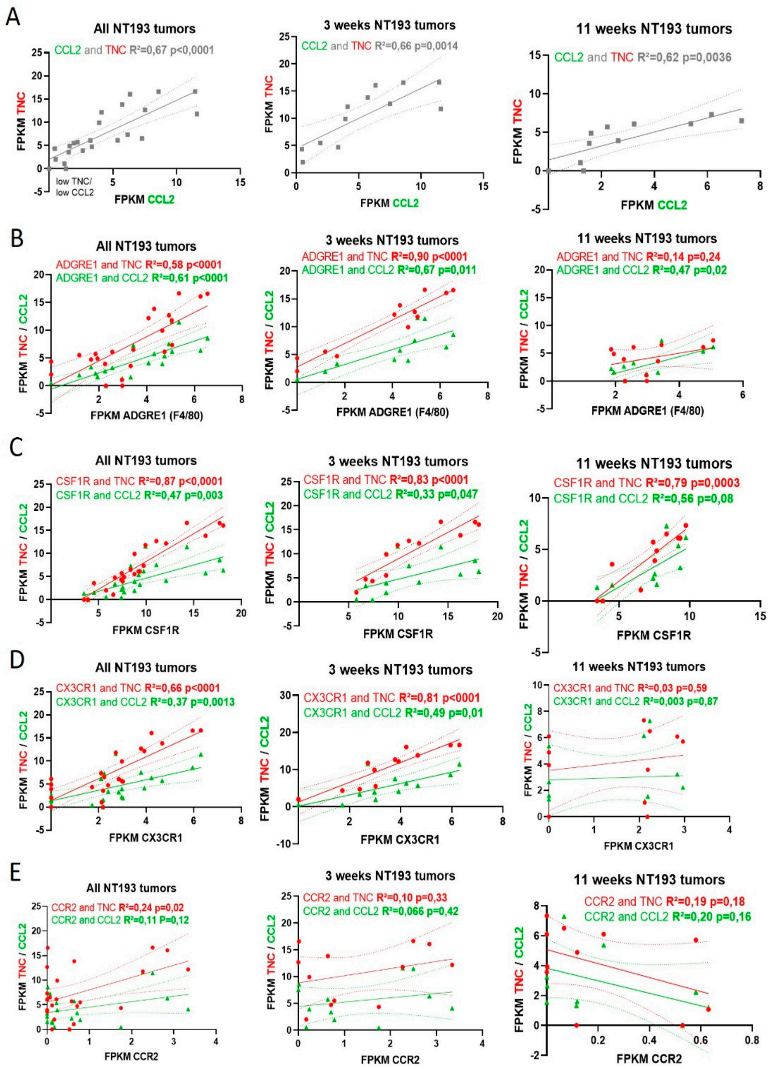

Supplementary figure S4: Linear regression curves analysis part 1: S4A) Linear regression curves indicating expression (in FPKM) of TNC in correlation to CCL2 in NT193 tumors at both 3 and 11 weeks tumors combined, 3 weeks and 11 weeks post-grafting. Linear regression curves indicating expression of (S4B) Adgre1, S4C) CSF1R, S4D) CX3CR and S4E) CCR2 in correlation to TNC (in red) and CCL2 (in green), respectively in all NT193 tumors (3 and 11 weeks old tumors together) or in only 3 weeks and 11 weeks NT193 tumors. P-values were determined by Pearson and Spearman tests in all NT193 tumors and in separated 3 and 11 weeks NT193 tumors, respectively.

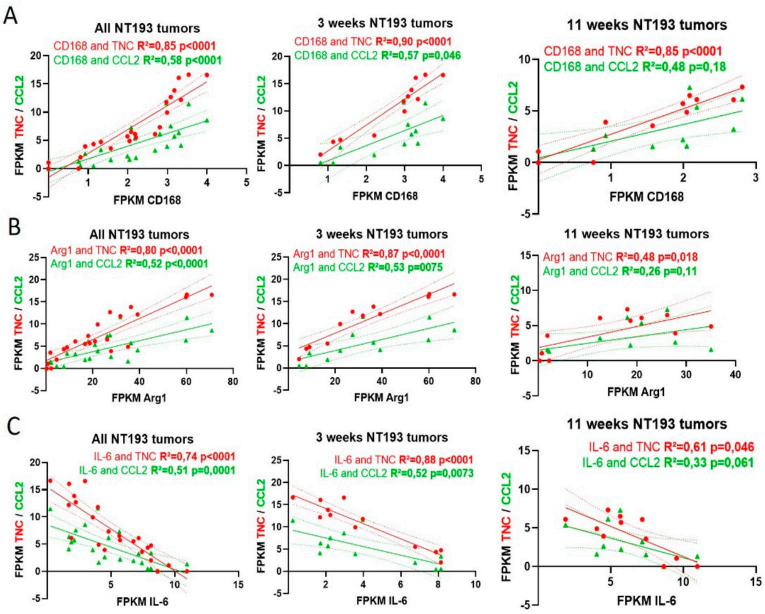

Supplementary figure S5: Linear regression curves analysis part 2: S5A) Linear regression curves indicating expression (in FPKM) of CD168, S5B) Arg1 and S5C) Il-6 in correlation to TNC (in red) and CCL2 (in green), respectively in all NT193 tumors or 3 weeks and 11 weeks separated NT193 tumors. P-values were determined by Pearson and Spearman tests in all NT193 tumors and in separated 3 and 11 weeks NT193 tumors, respectively.

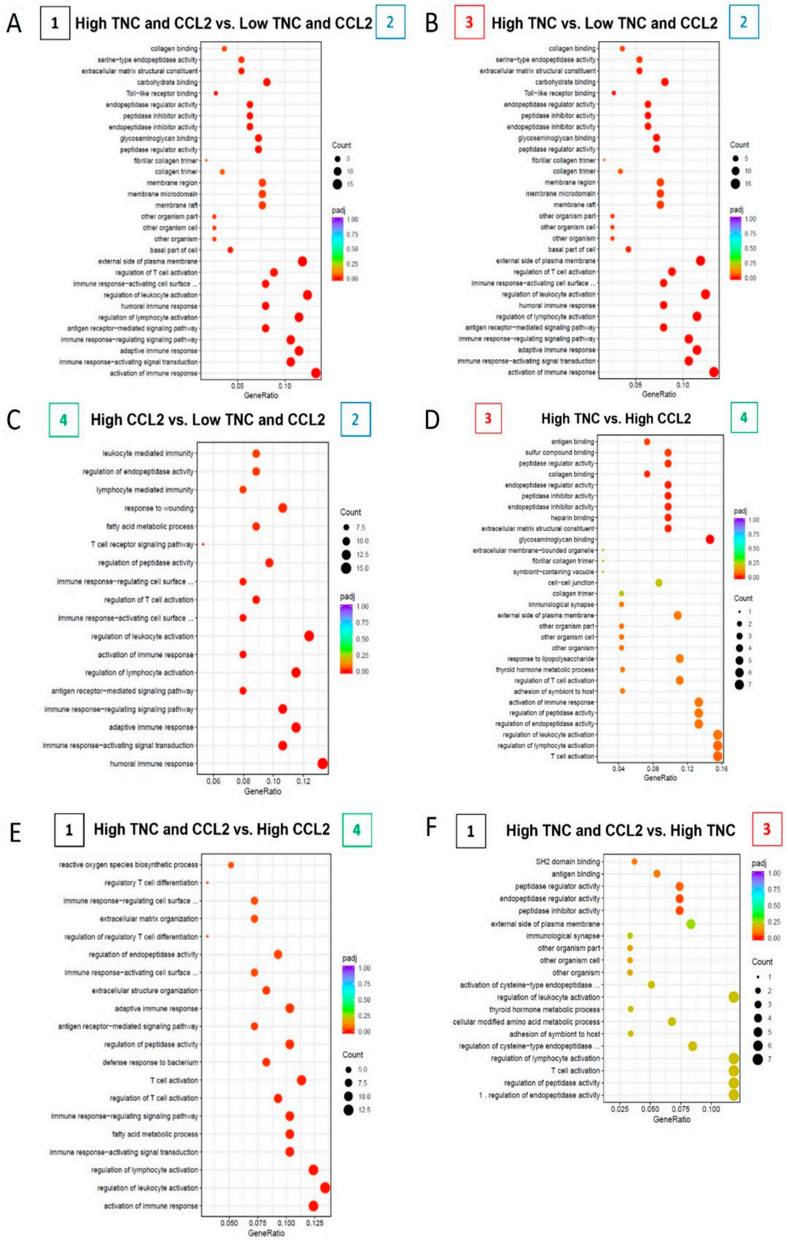

Supplementary figure S6: Gene ontology representation: Gene set enrichment analysis (GSEA) of the differentially expressed genes in S6A) high TNC and CCL2 tumors compared to low TNC and CCL2 tumors, S6B) in high TNC and low CCL2 tumors compared to low TNC and CCL2 tumors, S6C) in low TNC and high CCL2 tumors compared to low TNC and CCL2 tumors, S6D) in high TNC and low CCL2 tumors compared to low TNC and high CCL2 tumors, S6E) in high TNC and CCL2 tumors compared to low TNC and high CCL2 tumors, S6F) and in in high TNC and CCL2 tumors compared to high TNC and low CCL2 tumors. Enriched pathways and over-represented gene sets were selected from Gene ontology database for biological processes, molecular functions and cellular components.
